# Statement for electrophysiological procedures under the COVID‐19 pandemic from the Japanese heart rhythm society task force

**DOI:** 10.1002/joa3.12419

**Published:** 2020-09-07

**Authors:** Kazuhiro Satomi, Eiichi Watanabe, Seiji Takatsuki, Seiji Fukamizu, Yu‐ki Iwasaki, Daiji Takeuchi, Akihiko Nogami

**Affiliations:** ^1^ Heart Rhythm Center Tokyo Medical University Hospital Tokyo Japan; ^2^ Department of Cardiology Fujita Health University Bantane Hospital Nagoya Japan; ^3^ Department of Cardiology Keio University School of Medicine Tokyo Japan; ^4^ Department of Cardiology Tokyo Metropolitan Hiroo Hospital Tokyo Japan; ^5^ Department of Cardiovascular Medicine Nippon Medical School Tokyo Japan; ^6^ Department of Pediatric Cardiology and Adult Congenital Cardiology Tokyo Women’s Medical University Hospital Tokyo Japan; ^7^ Department of Cardiology Faculty of Medicine University of Tsukuba Tsukuba Japan

**Keywords:** COVID‐19, electrophysiology, pandemic, practice, recommendations

## Abstract

COVID‐19 is a global catastrophe with markedly reduced health and economy of human civilization. Heart rhythm disorder has also been impacted by this disease. This statement is the universal criteria for EP procedures in the new era, which we will face during COVID‐19 pandemic. We described the methods of triage based on the severity of disease, the regional state of pandemic and supply of medical resources. This guidance will be the universal criteria for EP procedures in the new era, which we will face during and after the COVID‐19 pandemic.

## Graphical abstract

This statement of Japanese Heart Rhythm society was published to address the issues faced by electrophysiologists and to provide standard indications for the regulation and advancement of EP procedures for health care professionals during and after the COVID‐19 pandemic. We described the methods of triage based on the severity of disease, the regional state of pandemic and supply of medical resources. This guidance will be the universal criteria for EP procedures in the new era, which we will face during and after the COVID‐19 pandemic

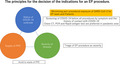

The severe acute respiratory syndrome coronavirus 2(SARS‐Cov‐2) is responsible for COVID‐19 and has a broad spectrum of presentations ranging from asymptomatic disease to severe respiratory disorder, myocardial injury and death. Up to 20%–30% of patients hospitalized with COVID‐19 have myocardial involvement. It may be caused by direct myocardial injury and thrombotic disease. The presence of cardiovascular injury related to worse prognosis in patients with COVID‐19. The management of cardiovascular injury is important to improve the prognosis of COVID‐19, including antiarrhythmic therapy and antithrombotic therapy.^1^

The global outbreak of COVID‐19 started in early 2020 in China and has quickly spread to the rest of the world. Many countries have been significantly impacted by this pandemic with thousands of deaths and these numbers will continue to worsen. ^2^

From the end of March, the number of patients diagnosed with COVID‐19 has also increased in Japan. At the end of May, 17 000 patients were diagnosed with COVID‐19 and 900 of those patients died. The Japanese government declared a state of emergency on April 4th and imposed a self‐confinement of daily and business activities. ^3^

The Japanese Heart Rhythm Society (JHRS) issued the first announcement on April 2, 2020. This announcement recommended the postponement of all elective electrophysiological (EP) procedures until the easement of the COVID‐19 pandemic. This announcement aimed to preserve the medical resources, such as ICU beds and personal protective equipment (PPE), and to avoid the exposure of patients and health care professionals to SARS‐CoV‐2.^4^, ^5^

With the improvement in the number of infections and the health care system, the Japanese government lifted the state of emergency on May 25th. This has provided the opportunity to reintroduce the regular cardiovascular care in a progressive manner with appropriate safeguards.

The purpose of this statement from the JHRS is to address the issues facing electrophysiologists and to provide standard indications for the regulation and advancement of EP procedures for health care professionals during and after the COVID‐19 pandemic.

## 1 SURVEILLANCE OF THE COVID‐19 STATUS IN JAPANESE EP CENTERS

A questionnaire was sent out to the 417 EP centers to elucidate the situation of the management of COVID‐19 in the EP centers and shortage of medical resources, such as masks and surgical gowns. Google Forms was used for a web‐based questionnaire investigation from May 11 to 18, 2020. The questionnaire included, 1) the number of catheter ablation and device implantation cases from June to April 2020, 2) the postponement and limitations of the daily practice of cardiology, 3) worsening cases during the postpone or limitations of EP procedures, 4) COVID‐19 screening in EP centers, and 5) the shortage of PPE supplies.^6^

**Figure 1:**
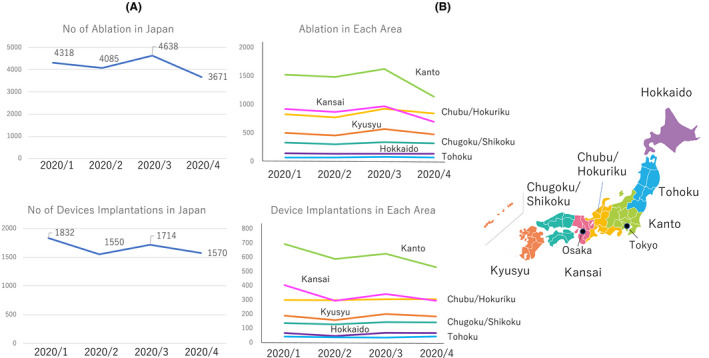
The number of Ablation and device implantations in Japan (A) and each area (B) from January 2020 to April 2020

The questionnaire results were obtained from 218 EP centers (52% of certificate EP training hospitals) from all over Japan. There was a regional difference in the number of procedures according to the burden of the pandemic. The number of EP procedures decreased according to the number of COVID‐19 cases in large cities, including Kanto (around Tokyo) and Kinki (around Osaka) areas. (Figure 1).

Eighty percent of the EP centers provided medical treatment for COVID‐19 cases and 75% of the centers postponed all elective EP procedures. Importantly, 8.5% of the centers experienced worsened patient conditions as a result of postponing the EP procedures, which was mainly development of heart failure as a result of atrial fibrillation. Screening tests were performed in 48% of the centers by Reverse Transcription (RT)‐PCR. RT‐PCR was routinely performed before admission in 9.1% of patients regardless of the symptoms, and 52.3% of the centers performed a symptom‐based RT‐PCR. The shortage of PPE was serious in many hospitals. The N95 masks and surgical masks were used for more than 1 day in 53% and 29% of all centers, respectively. The shortage of surgical gowns for EP procedures was also observed in 45% of centers regardless the region.

This surveillance demonstrated: 1) the number of EP procedures decreased in April 2020, mainly because of the surge in COVID‐19 cases, but had regional difference, 2) worsening heart failure was observed in patients with atrial fibrillation after postponing their elective catheter ablation, 3) in‐hospital PCR tests were only available in half of the EP centers, and 4) the PPE shortage is still serious even in the nonpandemic area in Japan.

## 2 REINTRODUCTION OF EP PROCEDURES

**Figure 2:**
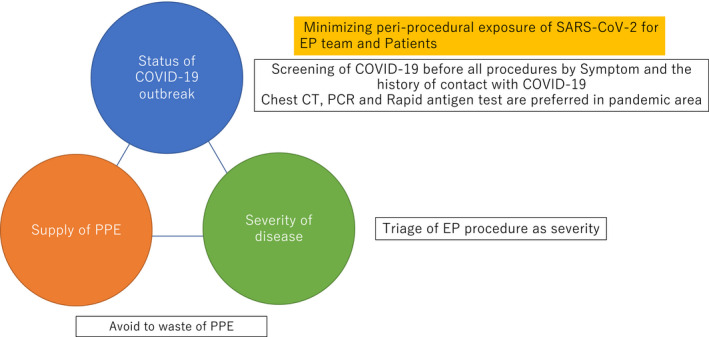
The principles for the decision of the indication for an EP procedure

Physicians should determine the indication for an elective EP procedure while considering three principle factors: 1) the regional burden of the COVID‐19 pandemic, 2) the PPE supply level, and 3) severity of the arrhythmias (Figure 2).

**Figure 3:**
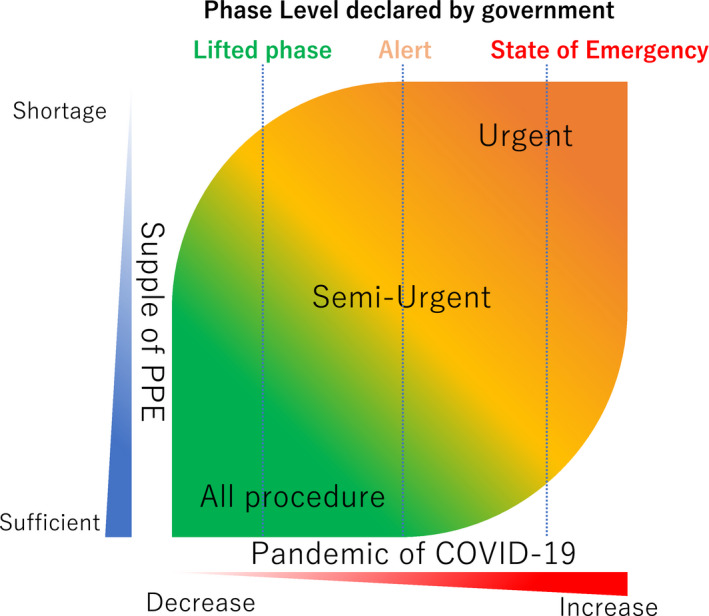
Triage during the EP procedure. See text for details

Some regions have seen an escalation in COVID‐19 cases when social restrictions have been eased. The healthcare providers should follow the updated information on the local status of the pandemic. There should be a rereduction in the EP procedures according to the rate of new COVID‐19 admissions and deaths in the relevant geographic area (Figure 3).

The use of PPE will be an important consideration during routine EP procedures. The PPE supply is not adequate in many facilities regardless of the status of the COVID‐19 outbreak in each region. The need to ensure the safety of the staff must be balanced against the need to conserve PPE supplies in the event the pandemic escalates.

The guidance of the Heart Rhythm Society stated that EP procedures should be triaged according to the severity of the disease.^7^ This guidance recommends that the electrophysiologist decide the EP procedure according to the severity of the disease while considering the balance between the status of the pandemic and PPE supply level. This guidance will be the universal criteria for EP procedures in the new era, which we will face during and after the COVID‐19 pandemic.

## 3 PROTECTION OF PATIENTS AND HEALTH CARE WORKERS DURING EP PROCEDURES (FIGURE 4)

- Prior to performing procedures in patients from both the in‐ and out‐patient settings, screening for COVID‐19 should be performed including an evaluation of the symptoms, physical examination, and history of contact with COVID‐19 patients.
- COVID testing (RT‐PCR or rapid antigen test) is recommended in all patients with an understanding that there may be false negative results. It is also important to identify ahead of time and ensure there are sufficient standard PPE for the procedures, as hospital resources can diminish quickly.
- Patients should wear masks during their hospital stay. A minimally invasive procedure with a shorter length of stay is preferable.
- If the patient is not intubated, a mask is placed on the patient prior to transport and during procedure.
- Transesophageal echocardiography has the potential risk of aerosol transmission of SARS‐CoV‐2. Alternative approaches to exclude LA (Left atrium) thrombi, such as enhanced cardiac CT (computed tomography) or intracardiac echo cardiograph should be considered before atrial fibrillation ablation according to the thromboembolic risk of patients.
- If intubation is required prior to the procedure, consider performing the procedure in a negative pressure room.
- PPE for patients with COVID‐19 or suspected cases
  - Airborne precautions should be taken, including N95 masks, surgical gowns and gloves and protective eyewear (goggles or face shield).
- PPE for patients negative for COVID‐19
  - Droplet precautions are reasonable.
  - Gowns and protective eyewear should be worn during the insertion of esophageal temperature monitoring catheters or airways.

## 4 CONCLUSIONS

Currently, there is no absolute treatment for this virus and several new treatments are being studied in many ongoing trials. ^8^

We are still facing a future explosive increase in COVID‐19 cases in Japan. On the other hand, we should continue providing a high standard of medical care for all heart rhythm disorders.

## DISCLOSURE

Authors declare no Conflict of Interests for this article.

5

**Figure 4:**
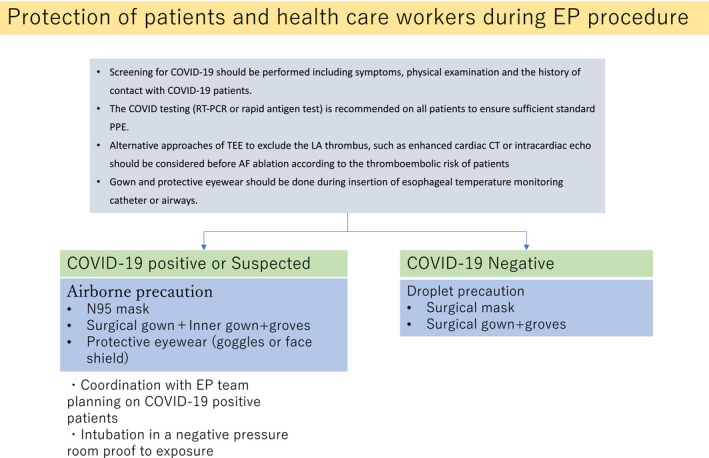
Protection of the patients and health care workers during EP procedures
